# Mississippi Valley Medical Association

**Published:** 1888-08

**Authors:** J. Lucius Gray

**Affiliations:** Secretary


### MISSISSIPPI VALLEY MEDICAL ASSOCIATION.

The Mississippi Valley Medical Association meets at St. Louis September 11, 12 and 13. The programme includes many papers and discussions of importance. The first day will be given to the discussion of abdominal surgery; the second, to infant feeding and some obstetric subject; the third day will be taken up with volunteer papers and some neuralogical subject. Arrangements for reduced rates are being made, and the society cordially invites all members of the profession in the Mississippi Valley to be present. J. LUCIUS GRAY, Secretary.

